# The Value of Signaling an Orthopaedic Surgery Program: A Survey to Orthopaedic Surgery Programs

**DOI:** 10.5435/JAAOSGlobal-D-23-00050

**Published:** 2023-06-02

**Authors:** Jacob C. Sorenson, Patrick M. Ryan, Russell A. Ward, Douglas S. Fornfeist

**Affiliations:** From Baylor Scott and White Orthopaedic Surgery Residency, Temple, TX.

## Abstract

A new system was implemented by the Association of American Medical Colleges called the preference signaling program for the 2022 to 2023 orthopaedic surgery residency match. Applicants were able to signal 30 orthopaedic surgery programs to indicate high interest in a specific program. The purpose of this study was to address how important signaling was to an orthopaedic surgery program this 2022 to 2023 application cycle.

**Methods:**

A five-question survey was sent to orthopaedic surgery residency programs participating in the Electronic Residency Application Service this application cycle. Contact information was gathered through the Accreditation Council for Graduate Medical Education residency website and program websites.

**Results:**

Responses were obtained from 69 of the 151 programs (46%) contacted. The average number of applicants per program was 727 (range, 372 to 1031, SD 155). Thirty-four of 61 respondents (56%) stated that 100% of their interviewees signaled their program. Fifty-five of 61 respondents (90%) indicated that their interviewee pool consisted of 75% or more applicants who signaled. Applicants who signaled had a 24.4% (range, 12.77 to 47.41, SD 8.04) chance of receiving an interview. Applicants who did not signal had just a 0.92% (range, 0 to 13.10, SD 2.08) chance of receiving an interview. Fifty-four of the 63 applicants (86%) answered that signaling played an important role in considering an applicant for an interview.

**Conclusion:**

Over half of the responding programs only interviewed applicants who signaled their program, and over 90% of programs' interview lists consisted of at least 75% of signaling applicants. Eighty-six percent of programs indicated that signaling played an important role in considering an applicant for an interview. Applicants who signaled were 26.5 times more likely to receive an interview than those who did not (*P* < 0.0001). With this information, applicants can narrow down their list of programs to apply to, knowing that their signal to a program will give them a better chance at receiving an interview.

Orthopaedic surgery applicants consistently apply to more programs than any other specialty in medicine. In the 2021 to 2022 application cycle, applications increased from 72 to 90, a 25% increase from the previous year.^1^ With the new implementation of the preference signaling program (PSP) from the Association of American Medical Colleges for orthopaedic surgery programs, applicants are now able to “signal” 30 programs.^2^ This allows programs to know that an applicant has ranked them higher on their list of programs in which they want to interview.

Orthopaedic surgery continues to be a highly sought-after field in medicine and continues to increase in competitiveness each year.^3^ Understanding how to better narrow down the applicant pool has been a topic of interest over the years with different variations of how to score and rank interviewees.^4,5^ To date, very little emphasis has been placed on helping minimize the increasing application phenomenon, with no systems in place to guide students to find the best programs for them. A recent survey showed that program directors of orthopaedic surgery programs ranked signaling the third most important factor in receiving an interview, only behind away rotations and personal knowledge of the applicant.^6^ This new PSP may be a simple and effective way to narrow down an applicant's list of programs to apply to, if signaling proves to be a useful way to show interest in a program and further increase their likelihood of receiving an interview.

In this study, a survey of programs participating in the PSP would help both programs and students alike by knowing whether this is a useful tool for programs to know which applicants have high interest in their program and for students to show interest in their most desirable programs. The primary purpose of this study was to survey program directors and coordinators to evaluate whether signaling in this 2022-2023 application cycle was an effective method to receive an interview from a program. The secondary purpose of this study was to evaluate whether signaling played an important role in deciding whom to interview from a program’s perspective.

## Methods

In total, 200 orthopaedic surgery residency programs were found to be participating in the 2022 to 2023 Electronic Residency Application Service and the National Residency Matching Program. Of the 200 programs, 90 programs publicly listed their program director's e-mail and 151 programs publicly listed a program coordinator's e-mail. If a program had both the e-mails of the program director and the program coordinator listed, an e-mail was sent to both. A five-question survey was issued to 151 programs in total. The survey consisted of three main components: the percentage of applicants who signaled the program, the percentage of interviewees who signaled the program, and a yes/no question regarding the significance of signaling in interview consideration. All responses were recorded through SurveyMonkey (https://www.surveymonkey.com/r/VN5VF8D) in an anonymous fashion. The survey is available for review in Supplement 1. Data were analyzed using data analytics software built into Microsoft Excel. Fractions were rounded to their nearest whole number to represent applicants. Fractional numbers were carried to two decimal places when not representing applicants. A Student *t-*test was used to calculate the *P* value associated with the mean percentage chance of receiving an interview.

The survey was issued on December 5, 2022, and response data were collected until December 23, 2022. A follow-up e-mail was sent on December 14, 2022, and December 20, 2022. This research did not receive any specific grant from funding agencies in the public, commercial, or not-for-profit sectors. All information used in this study was freely available on orthopaedic residency program websites, and institutional review board exempt approval was granted (Institutional Review Board #384688).

## Results

Responses were obtained from 69 of the 151 programs (46%) contacted. Six programs indicated that they were not participating in the signaling process and, therefore, declined to submit responses. There were 61 responses to the interviews section, 60 responses to the applications section, and 63 responses to the signaling significance section.

The average number of applicants per program was 727 (range, 372 to 1031, SD 155). The average number of applicants who signaled a program was 261 (range, 98 to 531, SD 93). Thus, approximately 36% of applicants to a program had signaled that program (range, 15% to 67%, SD 9%).

**Figure 1:**
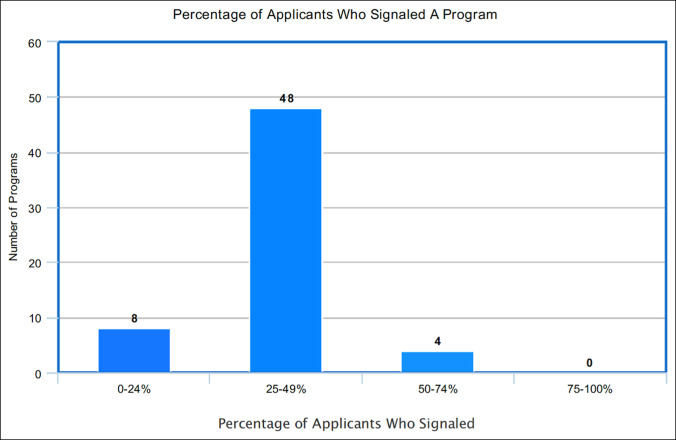
Chart showing percentages of applicants who signaled a program.

**Figure 2:**
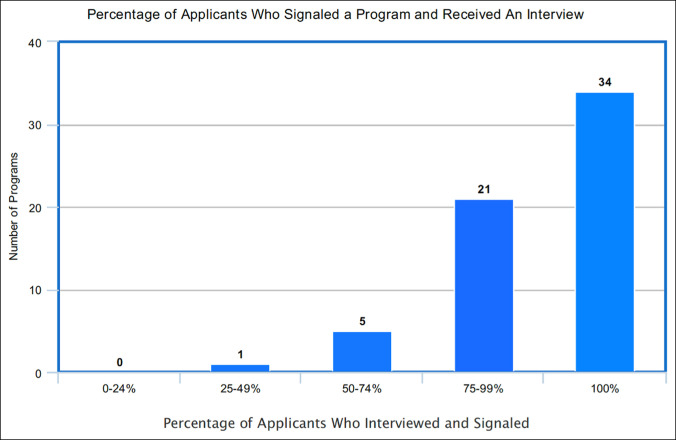
Graph showing percentage of applicants who signaled a program and received an interview.

The average number of interviews extended was 65 (range, 20 to 113, SD 23). Of the interviews extended, the average number of interviewees who signaled was 61 (range, 20 to 125, SD 24). The average percentage of interviewees who signaled the program was 93% (range, 41 to 100%, SD 13). Thirty-four of the 61 respondents (56%) stated that 100% of their interviewees signaled their program. Fifty-five of the 61 respondents (90%) indicated that their interviewee pool consisted of 75% or more applicants who signaled. Only one responding program reported that less than half of their interviewees signaled their program. For additional analysis, the resulting percentages were grouped by quartile and are presented in Figures 1 and 2 and Tables 1 and 2.

**Table 1:** Percentage of Applicants Who Signaled a Program and Received an Interview

No. of programs	0	1	5	21	34
Percentage of signaled applicants interviewing	0%-24%	25%-49%	50%-74%	75%-99%	100%

**Table 2:** Percentages of Applicants Who Signaled a Program

No. of programs	8	48	4	0	0
Percentage of signaled applicants interviewing	0%-24%	25%-49%	50%-74%	75%-99%	100%

**Figure 3:**
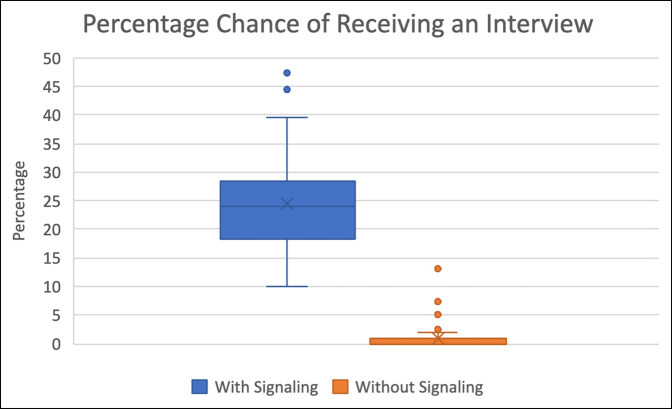
Graph showing percentage chance of receiving an interview.

On average, applicants who signaled had a 24.4% (range, 12.77 to 47.41, SD 8.04) chance of receiving an interview. Contrarily, applicants who did not signal had just a 0.92% (range, 0 to 13.10, SD 2.08) chance of receiving an interview. In other words, applicants who signaled were 26.5 times more likely to receive an interview than those who did not (*P* < 0.0001) (Figure 3).

**Figure 4:**
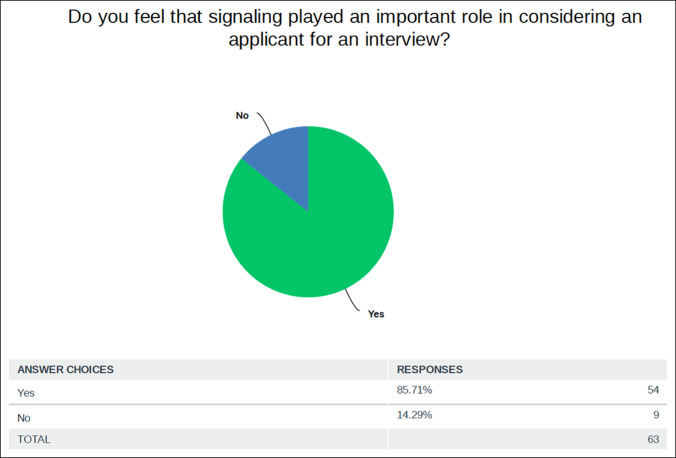
Graph showing percentage of programs who think that signaling was an important factor in extending an interview.

Finally, 63 responses were received to the binary question of importance of signaling during the interview process. Fifty-four of the 63 respondents (86%) answered that signaling played an important role in considering an applicant for an interview (Figure 4).

## Discussion

The most important finding of our study is that, on average, over 90% of orthopaedic interviewees signaled the program they were interviewing at. With the implementation of the PSP, the results show that programs heavily favored interviewing applicants who signaled their program. Over half of the responding programs only interviewed applicants who signaled their program, and over 90% of programs' interview lists consisted of at least 75% of signaling applicants. Only one program had an interviewee pool consisting of less than 50% of signaled applicants. This is consistent with the program responses to the importance of signaling because an overwhelming majority thought that signaling played an important role during their interview process. These data show that the importance of signaling a program is highly valuable for both orthopaedic surgery programs and applicants.

For the 2023 to 2024 application cycle, programs will have one less crucial statistic in their toolbox, a United States Medical Licensing Examination step 1 score. The US Medical Licensing Examination announced in 2021 that the first board examination for medical students would not have a numerical value.^7^ For many years, step 1 scores were considered the benchmark for filtering unqualified applicants in highly competitive fields. Orthopaedic surgery programs consistently ranked step 1 score as the most important factor when deciding whom to interview.^8^ With arguably the most important filter terminated, programs will need a new tool to help filter hundreds of applications received each year. A recent survey of program directors showed that a new emphasis for applicants will shift toward United States Medical Licensing Examination step 2 scores.^9^ The implementation of the PSP will also allow programs to have a new ability to easily gauge the interest of applicants in their program and potentially add a new filter in the application process. In the review of applications, there could be a role of subgrouping of applicants as “signaled” in addition to the rotating pool that could enhance the quality of the review process in an otherwise diluted process of review among so many applicants for programs.

Regarding the growing competitiveness of orthopaedic surgery residencies and increased applications, this new information about signaling shows that applicants are much more likely to interview at a program they signaled. Understanding this crucial information could potentially start a new shift toward diminishing applications to programs. Otolaryngology residency programs implemented a signaling system in the 2020 to 2021 cycle in which applicants could signal five programs. They found that signaling greatly increased the likelihood of receiving an interview when compared with not signaling a program (58% vs 14%, *P* < 0.001).^10^ Otolaryngology applications increased from 72.2 to 77.7 from the 2021 cycle to the 2022 cycle, but this is likely because of this study being published after the 2022 cycle had begun. Additional insight can be gathered with this coming 2023 application cycle and the potential effect of signaling in otolaryngology.^11^

For applicants, narrowing down the list of numerous orthopaedic programs is essential. The continuing trend of applying to more and more programs is not sustainable. Many factors go in to decide where an applicant will send an application, but as the desire to become an orthopaedic surgeon increases, the number of applications increases as well. This phenomenon is placing a burden on applicants to apply to more and more programs every year, likely to programs they would typically not send an application. The National Residency Matching Program applicant surveys consistently show that applicants rank programs based on their geographic preference.^12^ Allowing applicants to now signal 30 programs will help applicants express the real intent to train at a specific program in the geographic preference of their choice. Applicants on average complete 2.4 away rotations during their time in medical school, with the average cost of each rotation equating to $2,799.^13^ Away rotations can be considered the supreme way to show a program that one is interested and also to find a great fit, but with the addition of signaling, an applicant can now show a select few more programs their intentions.

This study does have limitations. Not all orthopaedic surgery programs participated in this study; thus, the views of those who participated may not represent the majority. In addition, our study did not analyze the effect of PSP in light of visiting student rotations, a potential confounding variable. Additional studies are needed to fully comprehend the effect of the PSP. In addition to further stratifying by visiting rotation participation, analyzing the effect of the PSP by geographic region would be useful. A postmatch analysis of signal preference will also be very useful to understand its role in the match process in addition to the presently analyzed interview process. This study shows that orthopaedic surgery residency programs are much more likely to interview an applicant who signaled their program, but the viewpoint of an applicant and their interview statistics based on the programs they signaled should be explored.

## Conclusion

Most of the programs (56%) offered an interview only to applicants who signaled their program. 86% of the programs also thought that signaling was an important factor in deciding who to interview this application cycle. Applicants who signaled a program were over 26 times more likely to receive an interview than those who did not. With this information, applicants can narrow down their list of programs to apply to, knowing that their signal to a program will give them a better chance at receiving an interview. Additional analysis is needed of the statistical outcomes from orthopaedic surgery applicants after this match cycle has been completed.
